# Instrument tables equipped with local unidirectional airflow units reduce bacterial contamination during orthopedic implant surgery in an operating room with a displacement ventilation system

**DOI:** 10.1016/j.infpip.2022.100222

**Published:** 2022-05-26

**Authors:** Josefin Seth Caous, Karin Svensson Malchau, Max Petzold, Ylva Fridell, Henrik Malchau, Linda Ahlstrom, Peter Grant, Annette Erichsen Andersson

**Affiliations:** a Department of Methodology, Textiles and Medical Technology, RISE Research Institutes of Sweden, Gothenburg, Sweden; b Department of Orthopedics, Institute of Clinical Sciences, Sahlgrenska Academy, University of Gothenburg, Sweden; c Department of Orthopedics, Sahlgrenska University Hospital, Gothenburg, Sweden; d School of Public Health and Community Medicine, Institute of Medicine, Sahlgrenska Academy, University of Gothenburg, Sweden; e Health & Care Sciences, Sahlgrenska Academy, University of Gothenburg, Sweden; f Harvard Medical School, Harvard University, Boston, MA, USA; g Lovisenberg Diakonale Hospital, Oslo, Norway

**Keywords:** Unidirectional airflow, Orthopedic surgery, Instrument table, Surgical instruments, Bacterial count, Colony forming unit

## Abstract

**Background:**

Airborne bacteria present in the operating room may be a cause of surgical site infection, either contaminating the surgical wound directly, or indirectly via e.g. surgical instruments. The aim of this study was to evaluate if instrument and assistant tables equipped with local unidirectional airflow reduce bacterial contamination of the instrument area to ultra clean levels, during orthopedic implant surgery in an operating room with displacement ventilation.

**Methods:**

Local airflow units of instrument and assistant tables were either active or inactive. Colony forming units were sampled intraoperatively from the air above the instruments and from instrument dummies. A minimum of three air samples and two-three samples from instrument dummies were taken during each surgery. Samples were incubated on agar for total aerobic bacterial count. The mean air and instrument contamination during each surgery was calculated and used to analyze the difference in contamination depending on use of local airflow or not. All procedures were performed in the same OR.

**Results:**

188 air and 124 instrument samples were collected during 48 orthopedic implant procedures. Analysis showed that local unidirectional airflow above the surgical instruments significantly reduced the bacterial count in the air above assistant table (*P*<0.001) and instrument table (*P*=0.002), as well as on the instrument dummies from the assistant table (*P*=0.001).

**Conclusions:**

Instrumentation tables equipped with local unidirectional airflow protect the surgical instruments from bacterial contamination during orthopedic implant surgery and may therefore reduce the risk of indirect wound contamination.

## Introduction

Surgical site infection (SSI) is a dreaded complication after implant surgery [[1], [2], [3]] often leading to resource demanding treatment and implant replacement [[4], [5], [6]]. Airborne contamination of the wound, either directly, or indirectly through air-contaminated instruments or implants, has been described as an important risk factor for SSI in orthopedic implant surgery [[7], [8], [9], [10]]. Factors such as ventilation system, number of door openings, persons present, size of the operating room (OR) and type of clothing affect airborne microbial contaminants during surgery [11,12].

Air quality can be determined by measuring colony-forming units (cfu) i.e. the number of particles carrying viable bacteria in a specified volume of air. Implant surgery should be performed in ORs with so called ultra-clean air, where the air quality is ≤10 cfu/m^3^ [13,14]. High efficiency particular air (HEPA) filtered unidirectional airflow (UDAF) over the sterile zone has been reported more effective than conventional ventilation systems in providing an ultra-clean environment. However, installing UDAF in an OR with conventional ventilation is complex, expensive, and not always possible due to height limitations. Instead, localized UDAF units may be a possible complement to reduce cfu/m^3^ in defined zones of the OR.

Local UDAF units directed over instrumentation tables and the operation field can reduce contamination to ultra-clean levels during urological laparotomies [15] and tables equipped with units providing local UDAF over the tables can achieve the same during neurosurgeries [16]. UDAF-table have also been reported to reduce airborne contamination during orthopaedic surgery simulations [17,18]. However, thorough evaluation, under real use conditions, of the UDAF-tables capacity to reduce cfu during orthopedic surgery has not been performed. We hypothesized that the UDAF-tables reduce air contamination to ultra-clean levels during real use in orthopedic surgery in an OR equipped with conventional displacement ventilation.

## Methods and materials

### Features of the operating room, ventilation system and UDAF tables

**Figure 1:**
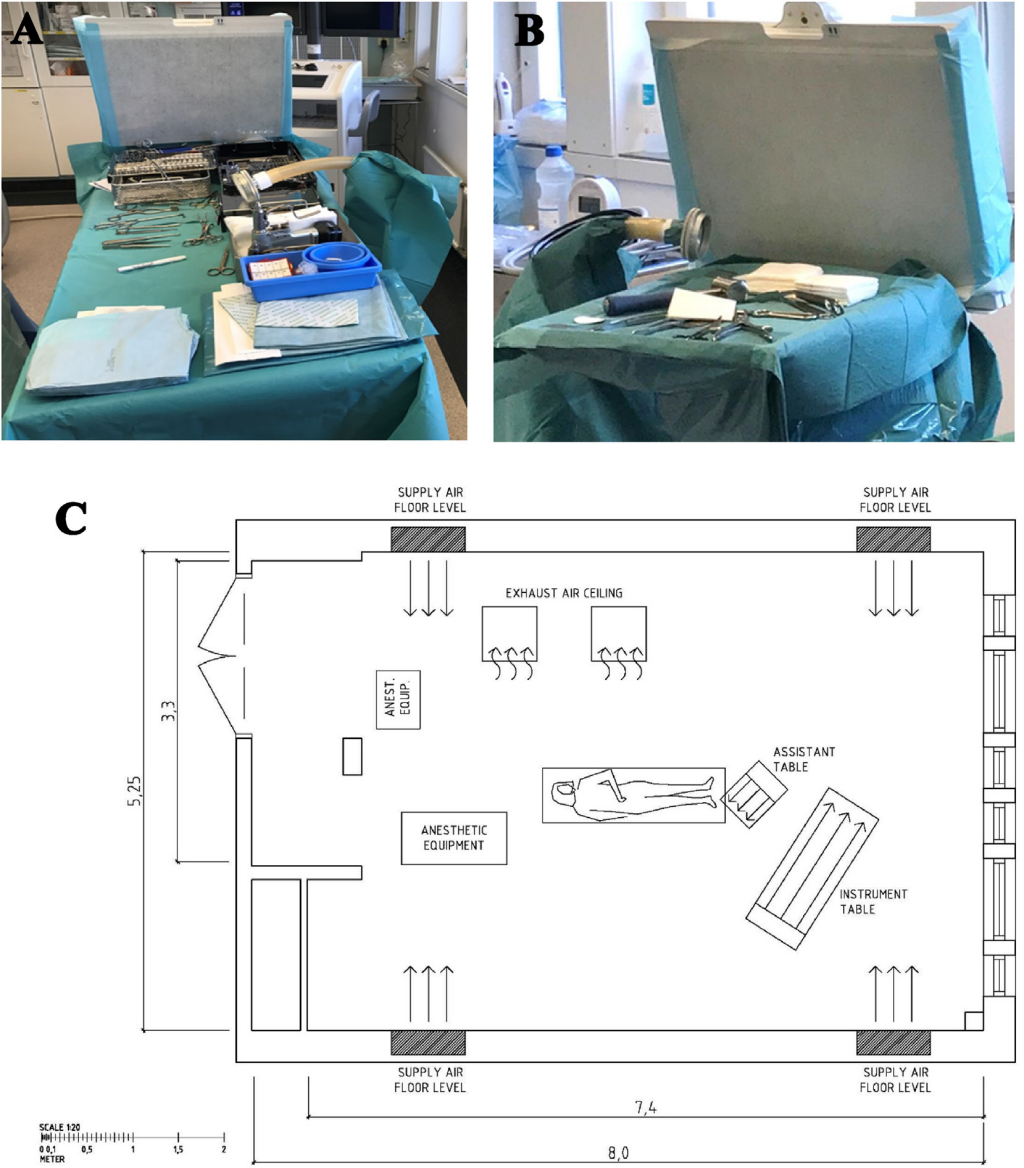
**Instrument (A) and assistant (B) tables with UDAF units at the end of the tables and the air sampler centered on the tables. The operating room (C) with conventional ventilation and instrumentation tables placed at the end of the operating table**. Instrumentation tables equipped with units creating a HEPA filtered horizontal unidirectional airflow (UDAF) over the surgical instruments were evaluated. Measurements were performed with the UDAF units either active or inactive. A holder for the air sampler was used, to enable reproduceable sampling at nearly the same location during every surgery. During the study only one active UDAF table, either instrument (A) or assistant (B) table, was used at a time. Example of the positioning of the tables relative the conventional ventilation of the operating room is shown in C.

All measurements were gathered in the same OR (net floor area 40.8 m^2^, air volume 121.7 m^3^) at the Sahlgrenska University Hospital, Mölndal, Sweden. The OR was equipped with conventional ventilation consisting of an air-displacement system, supplying HEPA (class H13) filtered air above the floor from each corner of the OR with the air outlets located in the ceiling (Figure 1). The ventilation system is controlled yearly and fulfills the recommendations of SIS-TS 39:2015 [14], has 21.8 air exchanges/h with a supply air flow of 560 L/s and exhaust air flow of 501 L/s. The SteriStay® instrument table and the Operio Mobile® assistant table (Toul Meditech AB, Västerås, Sweden) have UDAF units, attached at the end of the table, delivering ultra clean HEPA filtered air (at a velocity of 0.4–0.5 m/s or 400 m^3^/h) over the table surface, when active. The HEPA filter eliminates ≥99.9 % of the particles larger than 0.3 μm.

### Surgical procedures

Measurements were performed during orthopedic implant surgeries in the lower extremities (Table I). Surgeries due to infection, open fractures, or of too short duration to enable three air samples were excluded from the study. During each surgical procedure detailed information was recorded regarding type of procedure, surgical time, number of persons in the operating field, number of persons outside the operating field, number of door openings during each air measurement as well as during the entire procedure.

**Table I:** **Overview of surgery types during which measurements were sampled**. Measurements were performed during 55 surgeries, however measurements from 48 surgeries were included in the analysis. Surgery types where one surgery has been excluded, due to too short procedure time, ongoing infection, or growth on reference plates, are indicated with∗

	Ass. table UDAF on	Ass. table UDAF off	Inst. table UDAF on	Inst. table UDAF off	Inst. table conv.	Sum
Intramedullary nail	3	4	5∗	5∗	2∗	19
Plate osteosynthesis	4	2∗	4∗	4∗	3	17
Hemiarthroplasty	2	3	3	3	5∗	16
Other implants	1	2				3
Sum	10	11	12	12	10	55

### Surgical team and behavioural characteristics

The surgical team consisted of a chief surgeon, an OR nurse, a circulating nurse, an anesthetist nurse and frequently an assistant surgeon. In addition to the surgical team, the research assistant performing the measurements was present in the OR and occasionally also students and company representatives. All members of the surgical team and visitors wore conventional cotton/polyester OR clothes and hoods covering the hair, neck, and shoulders. The surgical team wore non-woven gowns (Mölnlycke® Barrier Surgical Gown, Classic), double sterile gloves and face masks. For each surgical team the adherence to dress regulations was recorded. Before initiating the study, the OR nurses at the department received training on preparing and using the UDAF instrumentation tables.

### Microbial sampling

Microbial samples, from air and instrument dummies (stainless-steel coupons, 4.3 x 4.3 cm), were collected during 55 orthopedic implant surgeries with the following instrumental tables: instrument table with active UDAF unit (n=12), instrument table with inactive UDAF unit (n=12), assistant table with active UDAF unit (n=10), assistant table with inactive UDAF unit (n=11) and conventional instrument table (n=10). The distribution of surgical procedures within each study condition is presented in Table I.

### Air samples

Air sampling was performed, according to SIS-TS 39:2015 [14] using a Sartorius MD-8 air scanner (Göttingen, Germany), sampling 1 m^3^ air through a sterile gelatin filter (3 mm pore size, 80 mm diameter), during 10 minutes. Three to four filters were collected during each procedure and placed on Columbia horse blood agar plates (Media Department, Clinical Microbiology Lab, Sahlgrenska University Hospital, Sweden). A holder for the filter unit ensured that the air sampling was performed at the center of the table at each surgery (Figure 1). Following SIS-TS 39:2015 [14], one reference plate per day was included as a batch control of the agar as well as a control of that the agar plates were not contaminated during transportation, in designated boxes, to the Clinical Microbiology Lab. The measurements were excluded from the study if less than three filters were obtained during one surgery or if growth occurred on the reference plate.

### Instrument dummy samples

The deposition of viable bacteria/cm^2^/hour onto instrument dummies placed on the instrument and assistant tables was measured. Deposited bacteria on the instrument dummies were transferred to Columbia horse blood agar plates for live count, after the end of the surgical procedure. The transfer was performed by placing the instrument dummies upside-down on the agar for approximately 10 minutes and thereafter removing them by gently tapping the closed plate so that the instrument dummy fell onto the lid and could be removed without risk of contaminating the sample. Three instrument dummies were distributed on the instrument tables, in the middle and at the ends, while two instrument dummies were positioned on the smaller assistant table, one at each end of the table. The instrument dummies were placed on the tables prior to surgery together with the rest of the instruments and covered with a sterile drape until start of surgery. The instrument dummies were collected by the OR nurse during closure of the wound, following hospital routines for handling of sterile material.

### Sample incubation and analysis

Agar plates were incubated aerobically for 48 hours at 35°C, following SIS-TS 39:2015 [14], and total aerobic bacterial count determined, at the Department of Clinical Microbiology, Sahlgrenska University Hospital, Gothenburg, Sweden.

### Statistical methods

From each surgery the mean contamination, measured as cfu/m^3^ in air and cfu/cm^2^/h on instrument dummies, was calculated. Descriptive data was generated using IBM SPSS® (Statistical Package for Social Sciences) version 25 and presented in Table II.

**Table II:** **Local UDAF units reduce cfu in air and surface samples taken from instrumentation tables during ongoing orthopaedic surgery**. The number of colony-forming units (cfu) was analysed in air and surface samples gathered from instrument and assistant tables equipped with unidirectional airflow (UDAF) units (either active or inactive during measurements) and conventional instrument tables. The mean and median of cfu in samples depending on condition is presented, as well as % of air samples fulfilling the recommendation of ≥10 cfu/m^3^ air. For instrument samples the limit was set to 0 cfu

Sample	Active UDAF unit	Inactive/no UDAF unit	*P*-value^a^
**Assistant table (Operio Mobile®)**			
No. of surgeries (air samples)	10 (40)	10 (38)	
Mean cfu/m^3^/surgery ± SD	0.3 ± 0.78	13.4 ± 13,25	
Median cfu/m^3^/surgery (min-max)	0 (0–2,5)	9,4 (2,8–45,5)	<0.001
Air samples with cfu ≤ 10	100 %	68 %	
No. of surgeries (instrument samples)	10 (20)	10 (20)	
Mean cfu/cm^2^/h/surgery ± SD	0.004 ± 0.127	0.052 ± 0.0476	
Median cfu/cm^2^/h/surgery (min-max)	0 (0–0.4)	0.035 (0-0,13)	0.001
Instrument samples with cfu = 0	95 %	45 %	
**Instrument table (SteriStay®)**			
No. of surgeries (air samples)	10 (39)	10 (39)	
Mean cfu/m^3^/surgery ± SD	0.2 ± 0.26	5.0 ± 2.77	
Median cfu/m^3^/surgery (min-max)	0.1 (0–0.8)	4.9 (1.3–10.8)	0.002^b^, <0.001^c^
Air samples with cfu ≤ 10	100 %	92 %	
No. of surgeries (instrument samples)	10 (30)	10 (30)	
Mean cfu/cm^2^/h/surgery ± SD	0.004 ± 0.0027	0.17 ± 0.022	
Median cfu/cm^2^/h/surgery (min-max)	0 (0–0.02)	0.015 (0–0.07)	0.1^b^, 0.08^c^
Instrument samples with cfu = 0	93 %	73 %	
**Conventional instrument table**	Not Applicable		
No. of surgeries (air samples)		8 (32)	
Mean cfu/m^3^/surgery ± SD		8.0 ± 1.52	
Median cfu/m^3^/surgery (min-max)		7.8 (2.8–14.0)	0.5^d^
Air samples with cfu ≤ 10		72 %	
No. of surgeries (instrument samples)		8 (24)	
Mean cfu/cm^2^/h/surgery ± SD		0.02 ± 0.022	
Median cfu/cm^2^/h/surgery (min-max)		0.015 (0–0.06)	0.9^d^
Instrument samples with cfu = 0		67 %	

UDAF, unidirectional air flow; cfu, colony forming unit; SD, standard deviation.

a Kruskal-Wallis nonparametric test, comparison between group.

b Comparison between instrument table with active or inactive UDAF.

c Comparison between instrument table with active UDAF and Conventional table.

d Compared to instrument table with UDAF turned off.

**Figure 2:**
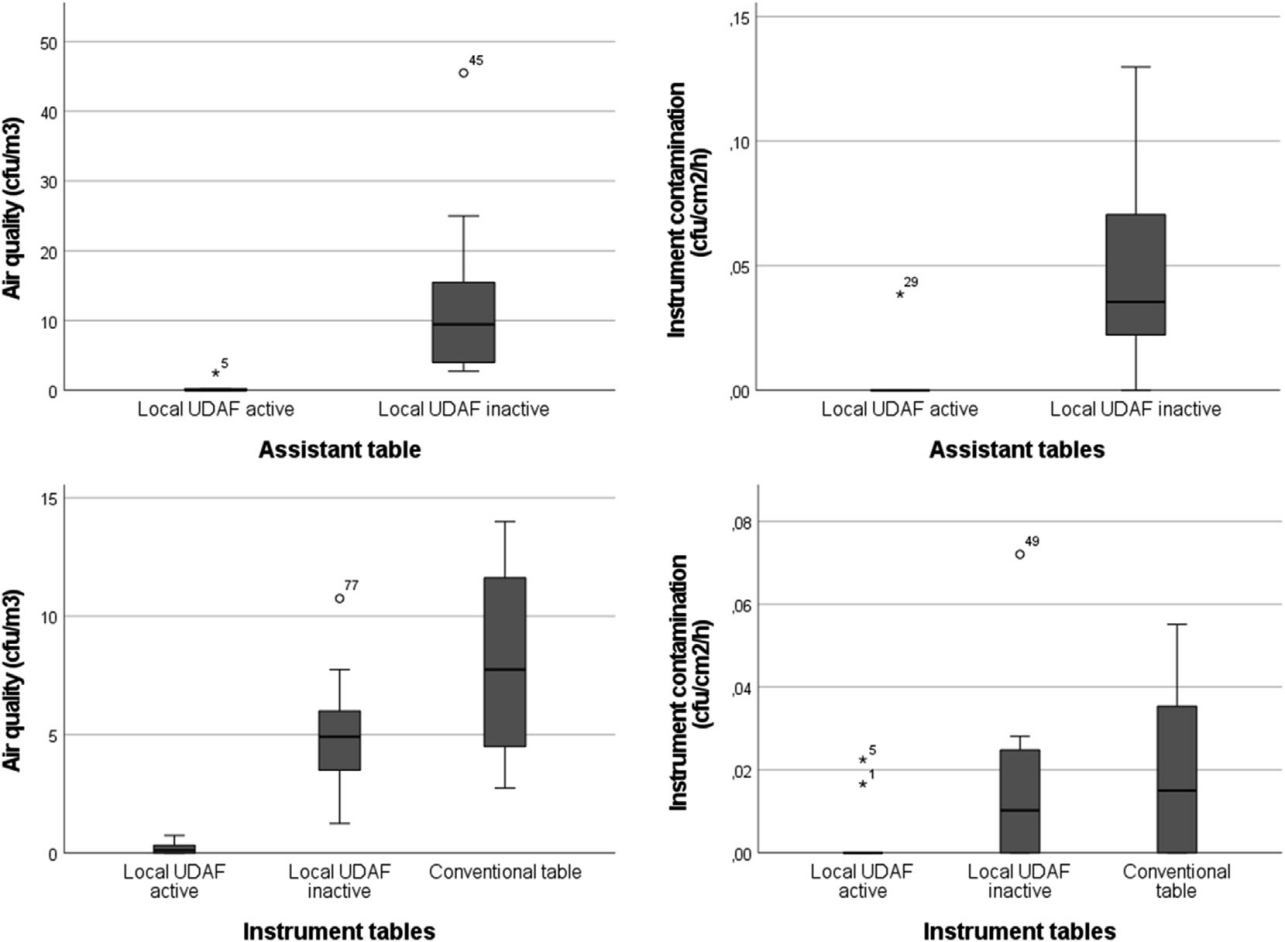
**Local UDAF units significantly reduced air and surface contamination around the surgical instruments during orthopedic implant surgery**. Results from active air sampling with a Sartorius MD-8 air scanner (left) and passive surface sampling with instrument dummies (stainless-steel coupons with a size of 4.3 x 4.3 cm) (right) at assistant tables (top row) and instrument tables (bottom row).

The outcome data was highly skewed, including a large proportion of zero values in the groups with active UDAF. This is a common problem when analyzing microbial samples where effective reduction is accomplished, and no optimal statistical method for this kind of data has been suggested. Nevertheless, we have chosen to perform a statistical analysis to complement the graphical presentation in Figure 2. First, differences in contamination distributions between different types of ventilation set-ups (instrument tables with active and inactive UDAF, assistant table with active and inactive UDAF and conventional instrument table) were tested using the Kruskal-Wallis nonparametric test comparison between groups (IBM SPSS®, version 25). Secondly, multiple linear regression was used to assess how contamination was affected by different types of ventilation set-ups, adjusted for number of people present in the OR and door openings, using the Stata version 17. Statistical significance was set to 5%.

## Results

A total of 211 air and 186 instrument dummy samples were collected during 55 orthopedic implant surgeries (Table I). Measurements from seven surgeries were excluded from the analysis (growth on the reference agar plate *n=3* (two surgeries shared the same reference plate), operation time less than 45 min *n=3,* and patient with ongoing infection in the surgical site *n=1*). After exclusion, 188 air samples and 124 instrument samples were included.

### Air samples

The number of cfu/m^3^ air ranged from zero to four at tables with active UDAF units and from zero to 60 (median nine) at tables with inactive or no UDAF unit (Table II). There were significantly less cfu in the air samples when the UDAF units were active compared to inactive, for both the instrument table (SteriStay®) (*P*<0.001) and assistant table (Operio Mobile®) (*P*<0.001) (Figure 2). There was no statistically significant difference in cfu in the air samples from the conventional instrument table and instrument table with inactive UDAF unit (*P*=0.2).

Of all air samples taken at tables with an active UDAF unit, 100 % fulfilled the specification of ultra-clean air [13,14]. Air samples from instrument tables of conventional design or with an inactive UDAF unit fulfilled the requirement in 72 % and 92 %, respectively. Air samples from the inactive UDAF assistant table fulfilled the requirement in 68 % of the samples (Table II).

### Instrument samples

Sampling of instrument dummies from the assistant table showed significantly lower cfu/cm^2^/h with active UDAF unit compared to inactive, corresponding with the results from air sampling. However, no statistically significant difference in contamination, depending on active or inactive UDAF unit, was seen in instrument samples from the instrument tables (Figure 2). Furthermore, instrument dummies placed on active UDAF tables remained contamination free throughout surgery to a greater extent (93–95%) compared to inactive UDAF tables and conventional tables (45–73%) (Table II).

### Association between number of persons in the OR, number of door openings and number of cfu in air and instrument samples

At each surgery the number of persons present varied from four to eight (median six) and the number of door openings varied from zero to 21 (median five). These factors did not affect the air quality in a multiple linear regression analysis (Table III). The statistically significant reduction of airborne cfu when local UDAF was used above the tables remained after adjusting for the number of persons and door openings (Table III). However, ventilation set-up had an independent significant effect in the model (*P*<0.03).

**Table III:** **Regression analysis of mean cfu in samples from instrument dummies and air, and association with local UDAF, number of door openings, and persons present in the OR**. Multiple linear regression was used to assess how air quality above the instrumentation tables as well as instrument dummy contamination was affected by number of door openings and number of persons present during surgery, as well as if local UDAF units were used or not

Compared factors	Air contamination			Instrument contamination		
	R-squared (adjusted)	Coefficient (95% CI)	*P*-value	R-squared (adjusted)	Coefficient (95% CI)	*P*-value
**Ass. table active vs inactive UDAF**	0.45 (0.34)			0.47 (0.37)		
- UDAF		-10.6 (-19.8–1.3)	0.03∗		-0.05 (-0.8–-0.01)	<0.01∗
- Door openings		-0.4 (-3.2–2.5)	0.8		<-0.01 (-0.02 – 0.00)	0.07
- Persons		4.2 (-1.2–9.6)	0.1		<0.01 (-0.02 – 0.00)	0.9
**Inst. table active vs inactive UDAF**	0.67 (0.60)			0.27 (0.13)		
- UDAF		-4.8 (-6.6–-2.9)	<0.001∗		-0.01 (-0.3–0.00)	0.1
- Door openings		0.4 (-0.3–1.1)	0.2		<0.01 (-0.00 – 0.01)	0.3
- Persons		0.06 (-0.8–0.9)	0.9		<0.01 (-0.00 – 0.01)	0.3
**Inst. table active UDAF vs Conv. Table**	0.72 (0.66)			0.25 (0.09)		
- UDAF		-7.6 (-10.7–-4.6)	<0.001∗		-0.01 (-0.03–0.00)	0.1
- Door openings		-0.5 (-1.7–0.7)	0.4		<-0.01 (-0.01 – 0.00)	0.5
- Persons		0.7 (-1.2–2.5)	0.5		<0.01 (-0.01 – 0.01)	0.9

CI, confidence interwall; ∗ Significant at 5% level.

Active UDAF reduced the amount of cfu sampled from instrument dummies on the assistant table (*P*=0.04) in a multiple linear regression analysis adjusted for number of persons and door openings. No statistically significant effect of UDAF, number of persons or door openings on cfu sampled from instrument dummies on the instrument table was found (Table III).

## Discussion

In the present study, instrumentation tables with local UDAF above the table surface significantly reduced the amount of cfu in the air above the instruments and on the surface of instrument dummies, during orthopedic implant surgery. A minimum of three air samples and two-three samples from instrument dummies were collected during each of the 48 included surgeries to get a representative mean of the air quality and instrument contamination during each surgery. To the authors knowledge, no previous study of this magnitude has been performed to evaluate the efficacy of UDAF tables during orthopedic implant surgery. The study thereby provides new insights in how air and instrument contamination is affected by use of UDAF tables.

The UDAF tables have a different design than conventional tables (Figure 1). To exclude this factor from the analysis the study was designed so that the results from active UDAF table were compared with results from the same table but with inactive UDAF. However, measurements on conventional tables were also included in the study.

The result from the present study of significantly reduced air contamination when using local UDAF, strengthens previously reported results from simulated use of UDAF tables during orthopedic procedures [17,18]. The results also cohere with reported cfu reduction using UDAF tables during neurosurgical procedures [16]. However, some differences in study design exist, von Vogelsang *et al.* performed measurements at several different locations during each surgery, including the surgical site area and the peripheral part of the OR, with mostly one-two samples per location [16]. Moreover, the UDAF assistant table could be placed so that the airflow was directed toward the surgical site, thereby reducing cfu levels at the surgical site as well. This is rarely possible in orthopedic implant surgery as the patient often needs to be accessed from both sides. Therefore, measurements at the surgical site were not included in the present study. Instead, the present study focused on the protection of instrument contamination and included sampling of instrument dummies as this gives an indication of cfu accumulating on the surgical instruments during surgery.

Although the method used in this study for measuring contamination on the surgical instruments is limited, it provides a good estimation of the difference in contamination depending on table used. Where 0 cfu was found in 93 % and 95 % of the samples taken from assistant and instrument tables with active UDAF units, respectively, compared to 45 % and 73 % of samples from the assistant and instrument tables with inactive UDAF units. Moreover, active UDAF units significantly reduced the cfu/m^3^ above assistant and instrument tables, so that 100% of the samples complied with the recommendations for clean surgery with ≤10 cfu/m^3^ [13]. Without the additional UDAF units, 68 % of the air measurements from the assistant table and 93 % from the instrument table met the recommended air quality. The higher number of cfu in the air and surface samples of the assistant table, compared to the instrument table, can be explained by the fact that the assistant table was positioned closer to the patient and staff than the instrument table. Therefore, the assistant table was exposed to more particles, generated from patient and staff, compared to the instrument table [12]. Nevertheless, when tables with active UDAF units were used, air contamination was below recommended values both above the assistant table and instrument table.

In order to analyze an association between cfu and events during the surgical procedure, the number of door openings during each air sample as well as entire surgery, and persons present were recorded for each surgery. The only factor that was found to significantly affect cfu level was if UDAF was used or not. Measurements at the surgical site have shown a correlation between air quality of the OR and number of door openings as well as number of persons present, in conventional ventilated ORs [19]. No such correlation was found when measuring above the instrument and assistant tables in the present study. The conflicting results could be due to the difference in measurement site.

### Conclusions

Assistant and instrument tables equipped with local unidirectional airflow (UDAF) units significantly reduced the number of cfu surrounding instrument and assistant tables to ultraclean levels during orthopedic implant surgery in an operating room (OR) with a conventional displacement ventilation system. Instrument and assistant tables equipped with UDAF units could therefore be a mean to reduce the risk of indirect bacterial contamination of the surgical wound in ORs with conventional ventilation systems. The reduction in wound contamination and the effect on infection rates are planned to be further evaluated.

### Limitations and risk of bias

The use of UDAF could not be blinded to the personnel due to the sound of the active UDAF units. Furthermore, the measurements were not performed in a randomized way, but for one condition at a time. However, after summarizing the different procedures performed during each condition, no major difference between groups were found (Table I).

The large proportion of zero values in the groups with active UDAF obstructed the statistical analysis. Methods to statistically handle tied observations are discussed in the literature [20]. However, all these methods have their pros and cons including the methods we applied. Thus, the statistical significances should only be seen as a complement to the descriptive graphical presentations.
